# The overwhelming use of rat models in nerve regeneration research may compromise designs of nerve guidance conduits for humans

**DOI:** 10.1007/s10856-015-5558-4

**Published:** 2015-08-22

**Authors:** Hilton M. Kaplan, Prakhar Mishra, Joachim Kohn

**Affiliations:** New Jersey Center for Biomaterials, Rutgers University, 145 Bevier Road, LSB-101, Piscataway, NJ 08854 USA

## Abstract

Rats are not the best model for the evolving complexities we face in designing nerve repair strategies today. The development of effective nerve guidance conduits for nerve regeneration is severely limited by the rat sciatic nerve model as the almost exclusive research model in academia. An immense effort is underway to develop an alternative to autologous nerve grafts for the repair of nerve defects, aiming particularly at larger gap repairs of 5–30 cm or more. This must involve combinations of ever more complex components, which in the vast majority of cases begin their testing in the rat model. Three major problems are at play: (1) The majority of nerve regeneration data is now being generated in the rat, which is likely to skew treatment outcomes and lead to inappropriate evaluation of risks and benefits. (2) The rat is a particularly poor model for the repair of human critical gap defects due to both its small size and its species-specific neurobiological regenerative profile. (3) Translation from rat to human has proven unreliable for nerve regeneration, as for many other applications. We explore each of these facets and their implications, in order to highlight the need for appropriate awareness in animal model selection when translating nerve regeneration modalities of ever-increasing complexity—from relatively simple devices to drug–device–biologic combinations.

## Introduction

Since 1880 researchers have worked on the development of alternatives to autologous nerve grafts for the repair of nerve defects [1]. This effort has been widely published over he past five decades, and has almost doubled over the past two: A PubMed search for “peripheral nerve repair” from 1995 to 2014 demonstrates a 1.8-fold decade-on-decade increase, from 1861 papers for the decade through 2004 to 3398 for the decade through 2014. As solutions for *short* nerve gaps have become available, the unmet need for the repair of *long* nerve gaps (gaps of 5–30 cm and more) has been recognized as a key research challenge. An alternative to nerve autograft repairs are nerve guidance conduits (NGCs), comprised of three components—a wall, a filler, and bioactive molecules—in an infinite number of potential variations and complexities. We believe that the development of effective NGCs, especially for large nerve gaps, is severely limited by the almost exclusive use of the rat sciatic nerve injury model in academia. Rats are not the best model for the evolving complexities we face, as three major problems are at play: (1) The preponderance of nerve regeneration data is now in a single species, which is likely to skew treatment outcomes and lead to inappropriate evaluation of risks and benefits. (2) The rat is a particularly poor model for the repair of human critical gap defects due to both its small size and its species-specific neurobiological regenerative profile: The real mismatch is that the rat model is used to test NGCs over 1–1.5 cm gaps, while they are ultimately supposed to work clinically in 5–30 cm gaps. (3) Translation from rat to human has proven unreliable for nerve regeneration, as for many other applications. We explore here each of these facets and their implications, in order to highlight the need for appropriate awareness in animal model selection when translating nerve regeneration modalities of ever-increasing complexity—from relatively simple devices to drug–device–biologic combinations. We propose that a serious lack of suitable research methods exists for addressing the real clinical need, which is the repair of larger nerve gaps; and that the vast majority of academic research is not adequately focusing on this clinical need. In an attempt to understand how translatable rat data has been for clinical use, and to inform our opinion presented here, the authors have reviewed the past 2.5 decades of English-language, in vivo and clinical nerve repair research.

Peripheral nerve injury is an increasing problem, in both incidence and severity, as populations increase together with concomitant increases in traffic and industrial accidents, violence, and battlefield injuries. Furthermore, the developing economies in many countries are producing larger pools of patients who have access to nerve regeneration therapies. It has been estimated that global nerve repair and regeneration costs will increase from $4.5 B in 2013 to $7.8 B in 2018 [2]. The predominant current treatment options involve autologous repairs: direct repair (for gaps of ~0–1 cm), nerve autografts (a substitute nerve from the patient; for gaps of ~1–10 cm), or vascularized nerve autografts (for gaps ~10+ cm) [3–5]. Despite being the gold standard, autografts are not as ideal as they may seem: they require an additional donor surgical site and procedure, with significant morbidity; there are limitations in terms of available length and size-matching; and donor nerves are usually sensory only, while the defects are usually mixed motor and sensory.

Alternatives have therefore evolved, including decellularized allografts (decellularized nerves from human cadavers, e.g., Avance^®^ (AxoGen, Alachua, FL)) [6]; vein conduits and other *biologic* NGCs, e.g., Neuragen^®^ (collagen Type I; Integra Life Sciences, Plainsboro, NJ); and *synthetic* NGCs, e.g., Neurotube^®^ (woven poly(glycolic acid) acid (PGA); Synovis Micro Companies Alliance, Birmingham, AL). Allografts overcome donor-site morbidity issues, but suffer from similar drawbacks to autografts and have poorer regenerative capabilities than autografts. Drawbacks of vein conduits and other biologic NGCs include harvest morbidity (for vein), collapsing, kinking, and lack of neurotrophic potential and/or promotion of scarring potential (e.g., components that promote fibroblast activity such as collagen). *Synthetic* NGCs may be regarded, ultimately, as the best possible approach; but as of now the simple materials used cannot match the performance of autografts and allografts, and offer only limited functional recovery. Therefore, to date only a very limited number of synthetic NGCs have obtained U.S. Food and Drug Administration (FDA) or Conformité Européenne (CE) approvals for clinical use (4 by 2014), and almost exclusively only for the repair of *short* gaps (≤4 cm; one ≤6.35 cm) [7, 8].

While the limitations recognized above drive the need for major innovations in the design of NGCs, two key scientific challenges need to be addressed before synthetic NGCs can match/surpass the regenerative outcomes seen with autografts: (1) *Chemical and mechanical properties* of the conduit wall need to be optimized, including parameters such as handling, suturability, stiffness, flexibility, compressive strength, and kink-resistance—particularly for use in longer nerve gaps, or for use across articulating joints such as the finger, elbow, shoulder, or knee. (2) *Effective biological enhancement strategies* have to be incorporated into synthetic NGCs, to create a biological milieu within the conduit that optimally supports and directs appropriate nerve regeneration.

Achieving NGCs that are capable of performing like autografts do, requires important innovations to each of the three main components of the NGC: the wall, filler and bioactive moieties. This involves testing numerous variants of each alone, and in combinations, to achieve polymers that support nerve regeneration; designs that are kink and compression-resistant while offering superior handling and suturability; and novel coatings and fillers that optimize mass transport (nutrient and waste exchange) while retarding the ingress of scar tissue. While in vivo studies are critical for pre-clinical testing, most academic laboratories almost exclusively use the rat sciatic nerve injury model, which is limited to about 1.5 cm in gap length, in protected anatomical sites that do not expose the NGC to significant mechanical forces or bending. Furthermore, rats (and particularly research-bred ones) have a strong regenerative potential and heal fast, in stark contrast to the compromised and diverse human patients that these NGCs will need to perform in.

## The preponderance of nerve regeneration data is in the rat

In order to gain insight into the degree to which nerve regeneration research relies on various animal models, the authors reviewed in vivo peripheral nerve repair studies, published in English over the last 25 years (Jan 1989–Sep 2014). Of 5723 PubMed references to peripheral nerve repair, 792 were short-listed as potentially relevant based on review of title and abstract (papers that were excluded at this stage were those that clearly had no surgical nerve repair component, e.g., mechanistic studies, in vitro work, etc.). Every short-listed paper was read and 284 of those were found to both have utilized a nerve grafting method for peripheral nerve repair in vivo, and to have met the following pre-established criteria: (1) nerve gap length was explicitly provided and was ≥0.5 cm (this excluded mouse models de facto; this criterion was implemented to exclude gaps over which NGCs would most likely not be needed clinically); (2) post-repair recovery was assessed quantitatively; (3) minimum follow up time after repair was 4 weeks. Rodent studies were included only if the minimum number of animals in each experimental group was five, however with larger animals such a criterion was not implemented due to the paucity of papers covering those models. The influence of nerve gap length on outcome after various types of nerve repair is still being analyzed, but of a total of 284 in vivo studies, 222 were with rats (78 %), 34 with rabbits (12 %), 11 with dogs, 6 with monkeys, 4 with sheep, 4 with cats and 3 with pigs. Rats and rabbits constitute 90 % of the data. Furthermore, the number of rat studies has demonstrated a marked increase, particularly since a notable inflection beginning in 2003—with an average of 4.8 (range 3–8) rat studies per year over the preceding decade, jumping to an average of 13.5 (range 6–26) per year over the decade since.

Windebank’s group performed a systematic review of animal models used to study nerve regeneration in tissue engineered scaffolds, and found similar numbers: 74.0 % of 416 studies having been performed in rat models, and 7.5 % in each of mouse and rabbit models [9]. That group noted that only three materials (collagen, polycaprolactone and poly(glycolic acid)) had progressed to clinical use following over 5 decades of research. Another conclusion was that a valid animal model was needed to accurately represent the specific processes that take place in *human* peripheral nerve regeneration. It was concluded that no distinct animal species met all the requirements for an ideal animal model. Certain models were considered well-suited for understanding regenerative neurobiology, while others were better for pre-clinical evaluation of efficacy. Their review identified that more than 70 synthetic materials had been tested across eight species, in 17 different nerves, over gaps of 1–90 mm, using over 20 types of outcomes, and without any standardization of methods between the publications. With regard to the rat model in particular, the authors recognized the benefits for which it has become so dominant: economy; relatively simple animal care; resistance to infection; feasibility of studying large groups; and relatively easy outcomes assessments (e.g., electrophysiology, functional recovery, muscle and nerve histology). They also noted a number of distinct disadvantages to this model: short gaps, that are particularly different from human gap lengths (the majority were 10 mm or less; range 1–50 mm); axotomies in rats that may undergo complete recovery, which is not the case in humans; peripheral nerve regeneration rates in rats that are faster than in humans; limited availability of genetic models and immunological probes for the rat; and the random use of different strains of rats (e.g., Lewis, Sprague–Dawley, etc.), without an understanding of differences in their regenerative potentials and responses to foreign materials. The authors emphasized an urgent need to standardize and/or rationalize animal models and outcomes methods for nerve regeneration studies.

Together with our literature review, these issues demonstrate a clear lack of suitable research methods for addressing clinical needs in peripheral nerve regeneration, and the progressively worsening potential for skewing of data.

## The rat is a particularly poor model for repair of critical gap defects

The rat is a particularly poor model for repair of critical and large gap defects, due to both its small size and its species-specific neurobiological regenerative profile. In the analysis described above, of the 284 in vivo studies collected, only 38 (13 %) reported nerve grafting across gaps ≥3 cm. These studies were performed in rat (8 of 222 rat papers had gaps ≥3 cm), rabbit (11 of 34 did), dog (7 of 11), monkey (3 of 6), sheep (4 of 4), cat (2 of 4) and pig (3 of 3) models.

This issue is further aggravated by the fact that healing within NGCs does not progress in a *linear* inverse relationship with gap length as one might expect. Consider that short gaps, that may take 1–2 weeks to heal, may in fact do better with empty conduits than those with fillers (due to the filler’s viscosity for example); whereas long gaps, that may take 6 weeks to heal, may do better with a filler (due to it’s architecture and Schwann cell friendly nature along which axons can regenerate; and the absence of any dead space for such a long duration). This has been demonstrated by Ezra and Kohn [Rutgers University, Piscataway, NJ; personal communication July 2015; in submission for publication].

The utility of animal models for gap length studies that are relevant to human defects is also confounded by variations in their neurobiological regenerative profiles [10], and the resultant differences that exist in critical gap lengths between species. A “critical nerve gap” is defined as a nerve gap over which no recovery will occur without some form of nerve grafting or bridging [7, 9]. In rats, the critical nerve gap is considered ~1.5 cm, in rabbits ~3 cm, and in pigs and humans ~4 cm. Although longitudinal rates of regeneration are preserved across vertebrate species at approximately 1 mm/day [11]—potentially due to the primitive and phylogenetically common ancestry of the neurite lengthening machinery involved—their critical gap lengths do differ. This is believed to be due, at least in part, to differences in the rates of fibrin-cable degradation within the NGC. Early on in the nerve repair process an acellular fibrin-extracellular matrix cable is laid down between the proximal and distal stumps [7, 12–14]. In rats this occurs within 1 week of repair, and is followed by cellular infiltration: Schwann cells, endothelial cells and fibroblasts migrate in along the cable from both the proximal and distal anastomoses. The Schwann cells proliferate and align to form highly aligned cables, the “glial bands of Büngner”, which provide neurotrophic and structural support for axonal regeneration. The fibrin cable is a critical component of this process, and due to variable thicknesses and degradation rates across species is believed to define the maximum gap length over which healing can occur. Its degradation time is approximately 2 weeks in the rat [15, 16], and up to 4 weeks in the human, leading to critical nerve gaps of ~1.5 cm in a rat sciatic nerve model and ~4 cm in humans.

Therefore, *simply performing longer repairs in rats cannot adequately model critical gaps in humans*. This has also been demonstrated in vivo. Vasudevan, et al. attempted to address the rat model’s limited utility for studying long nerve gaps due to their small size, by *looping* NGCs and isografts (genetically identical donor grafts, and so representative of an autograft) to create 3.5–4.0 cm gaps within a standard rat sciatic nerve transection model [17]. As may be expected from the fibrin cable mechanism described above, the 4 cm isograft group, and a 1 cm isograft control group, both showed robust regeneration in the distal nerve segment; while a 3.5 cm hollow NGC group failed to show any sign of nerve regeneration. We believe that, due to the neurobiological regenerative mechanisms described above, such models are flawed for comparison to human defects.

It is clear, therefore, that attempting to model critical gaps across species is not a reliable research technique: due not only to the simple inability to accommodate larger size NGCs, but also to differences across species in metabolic repair rates over different gap lengths. This strengthens our opinion that finding a NGC-filler combination that leads to *ideal* healing in an animal model over a human-sized critical gap of ~4 cm, would likely not perform the same way clinically.

## Translation from rat to human has proven notoriously unreliable

Translation from rat to human has proven unreliable for nerve regeneration [9, 18], as for many other applications [19–21]. There is a severe lack of suitable in vivo research methods for addressing the *clinical* needs of peripheral nerve regeneration. This is emphasized by the fact that while 5723 PubMed references describing peripheral nerve repair studies were published in English over the past 25 years, only 7 biologic and synthetic NGCs had received FDA/CE approval by 2014 [8]. While we recognize that much of this nerve research was not aimed specifically at NGC development, the shear disproportion of in vivo studies versus commercial products raises concerns that translation from rat to human has proven unreliable to date.

Tos, et al. provide guidelines for selecting appropriate animal models [18]. These highlight further important factors supporting our opinion that animal models do not approximate the clinical condition in many ways. We believe that some of the major reasons for this include: (1) healthy animals versus sick patients; (2) short versus long gap lengths (the clinical need for *large* gap repairs, while 90 % of in vivo studies are in rats and rabbits where gap lengths are usually ≤3 cm); (3) animal models that almost always employ *mixed sensory*-*motor* autografts for repairing mixed defects, versus clinical repairs that almost always involve *sensory* autografts (usually sural nerve) for repairing mixed defects; (4) protected anatomical sites in animal models, versus repairs that must often cross articulating joints in humans; and (5) inbred, highly homogeneous animal strains and ages, versus diverse patient populations and ages: It is well recognized that animal models fail to mimic the human condition in terms of the *uniformity* of animal subjects used. These models are designed this way to limit confounding variables. However, young, healthy, research-bred rats that have strong regenerative potential and rapid healing rates, provide a very different regenerative paradigm to that of wounded, compromised and diverse human patients (in terms of wound healing, vascularity, susceptibility to infection, etc.).

These issues are not unique to nerve regeneration studies. Based upon the retrospective re-examination of past clinical trials of more than 100 failed drugs, Hayden states that many drug candidates should have never made it to clinical trials. He suggests that the failure of promising experimental drugs could have been prevented with better animal studies [22]. This issue has been described with drugs for amyotrophic lateral sclerosis (ALS) [21], cancer [23], and analgesics [20], etc. Perrin reports that the positive results of candidate drugs for ALS seen in mouse trials were later found to be spurious, and probably due to poorly conducted studies [21]. He advocates for boosting the quality of animal studies through improved characterization and understanding of how rodent models correspond to human disease, as well as study designs that fight “noise”, such as excluding irrelevant animals, balancing for gender, splitting littermates among experimental groups, and tracking genes.

## Conclusions

We propose here that rats are not the best model to rely on for designing nerve repair strategies, particularly those that involve combinations of complex components designed to overcome large nerve gaps across mechanically challenging implant sites such as articulating joints.

We believe that the development of effective NGCs, especially for large nerve gaps, is severely limited by the almost exclusive use of the rat sciatic nerve injury model in academia. We emphasize the risks of assuming that 100 % of in vivo outcomes are clinically predictive, when 90 % of them have occurred in rat and rabbit models, with their inherent flaws as described here. This will have to change if we are to find solutions that can address the unmet clinical needs of repairing *long* nerve gaps on the order of 5–30 cm and more.

We propose limiting the use of the rat in nerve regeneration studies; and that this model should only be employed where background data strongly supports a model’s validity in answering highly specific questions around basic science. As advocated by Perrin, research towards improved characterization and understanding of how rat models correspond to human disease is essential, and techniques that fight “noise” should be employed [21].

An important question is raised as to how negatively the development of new technologies is impacted through the rat sciatic nerve model having become the essential “gate keeper”. We may be pushing technologies that show promise in the rat forward, while prematurely aborting the pursuit of those that fail in the rat, all before ever testing these in nerve injury models that are designed to mimic human clinical conditions more closely or more specifically.

We highlight the need for appropriate awareness in animal model selection when attempting to translate nerve regeneration modalities of ever-increasing complexity—from relatively simple devices to drug–device–biologic combinations.
